# The implementation and impacts of the Comprehensive Care Standard in Australian acute care hospitals: a survey study

**DOI:** 10.1186/s12913-024-11252-0

**Published:** 2024-07-11

**Authors:** Beibei Xiong, Christine Stirling, Daniel X. Bailey, Melinda Martin-Khan

**Affiliations:** 1 Centre for Health Services Research, The University of Queensland, Brisbane, QLD 4102 Australia; 2 School of Nursing, University of Tasmania, Hobart, TAS 7000 Australia; 3 Centre for Clinical Research, The University of Queensland, Brisbane, QLD 4102 Australia; 4 Department of Health and Life Sciences, University of Exeter, EX1 2HZ Exeter, England, United Kingdom; 5 School of Nursing, University of Northern British Columbia, British Columbia, V2N 4Z9 Prince George, Canada

**Keywords:** Comprehensive care, Care standard, Outcome measurement, Quality of care

## Abstract

**Background:**

Comprehensive care (CC) is becoming a widely acknowledged standard for modern healthcare as it has the potential to improve health service delivery impacting both patient-centred care and clinical outcomes. In 2019, the Australian Commission on Safety and Quality in Health Care mandated the implementation of the Comprehensive Care Standard (CCS). However, little is known about the implementation and impacts of the CCS in acute care hospitals. Our study aimed to explore care professionals’ self-reported knowledge, experiences, and perceptions about the implementation and impacts of the CCS in Australian acute care hospitals.

**Methods:**

An online survey using a cross-sectional design that included Australian doctors, nurses, and allied health professionals in acute care hospitals was distributed through our research team and organisation, healthcare organisations, and clinical networks using various methods, including websites, newsletters, emails, and social media platforms. The survey items covered self-reported knowledge of the CCS and confidence in performing CC, experiences in consumer involvement and CC plans, and perceptions of organisational support and impacts of CCS on patient care and health outcomes. Quantitative data were analysed using Rstudio, and qualitative data were analysed thematically using Nvivo.

**Results:**

864 responses were received and 649 were deemed valid responses. On average, care professionals self-reported a moderate level of knowledge of the CCS (median = 3/5) and a high level of confidence in performing CC (median = 4/5), but they self-reported receiving only a moderate level of organisational support (median = 3/5). Only 4% (n = 17) of respondents believed that all patients in their unit had CCS-compliant care plans, which was attributed to lack of knowledge, motivation, teamwork, and resources, documentation issues, system and process limitations, and environment-specific challenges. Most participants believed the CCS introduction improved many aspects of patient care and health outcomes, but also raised healthcare costs.

**Conclusion:**

Care professionals are confident in performing CC but need more organisational support. Further education and training, resources, multidisciplinary collaboration, and systems and processes that support CC are needed to improve the implementation of the CCS. Perceived increased costs may hinder the sustainability of the CCS. Future research is needed to examine the cost-effectiveness of the implementation of the CCS.

**Supplementary Information:**

The online version contains supplementary material available at 10.1186/s12913-024-11252-0.

## Introduction

Acute care hospital settings are characterised by a majority of patients with acute, serious or complex conditions [1, 2], necessitating a range of care services from multiple care professionals across disciplines and settings [3]. However, a lack of care coordination may result in fragmented care, which can lead to unnecessary hospitalisation, an increased length of stay in hospital, and adverse events [1, 4]. The traditional disease-specific approach to care delivery cannot meet the complex needs of patients resulting in a gradual shift by services to a more comprehensive care (CC) approach [5]. A rapid literature review identifying 16 articles on the effectiveness of CC indicated the potential of CC to improve health service delivery and positively impact both patient-centred care and clinical outcomes in acute care settings [6]. This literature review [6], conducted in 2015, was intended to inform Australian Commission on Safety and Quality in Health Care (ACSQHC) about the development of the Comprehensive Care Standard (CCS) in Australia.

Like most countries around the world, Australia’s population is aging, with people aged 65 and over increasing from 11% of the population in June 1992 to 17% in June 2022 [7]. This increase is associated with a significant increase in individuals suffering from chronic diseases, with approximately one-third of Australians self-reporting the presence of at least one long-term health condition in the 2021 Census [8]. The Australian health system is jointly run by three levels of government: local, state/territory, and federal (national) level [9, 10]. Whilst the Federal Government is responsible for developing national health policies and allocating money to the healthcare system, local and State Governments are responsible for implementing and delivering health services. Medicare is Australia’s universal health care scheme, which covers the costs of all public hospital services and some or all of the costs of other health services, including medical services provided by general practitioners and medical specialists and medicine.

In 2017, the ACSQHC released the CCS, one of the National Safety and Quality Health Service (NSQHS) Standards [11]. The CCS is intended to minimise the risk of patient harm and reduce adverse events, improve the safety and quality of care delivered, and ensure patients receive the total health care required or requested by them. The NSQHS standards are mandatory in all Australian hospitals. From 2019 hospitals are accredited against these standards. The accreditation assessment of the CCS is based on four criteria and 36 actions that constitute the CCS. The four criteria are: “1. clinical governance and quality improvement to support CC, 2. developing the CC plan, 3. delivering CC, and 4. minimising patient harm” [11]. As per Xiong et al.‘s (2023) review of national standards for CC, the development of a CC plan is consistently recognised as a fundamental requirement for achieving CC. While the specifics of such a plan may vary based on patient needs and healthcare settings, the Australian CC plan is required to adhere to the seven specific actions outlined within the CCS criteria for developing a CC plan.

According to the ACSQHC, CC is defined as the “coordinated delivery of the total health care required or requested by a patient” [11]. The ACSQHC has described a set of six essential elements for CC delivery: (1) clinical assessment and diagnosis, (2) identify goals of care, (3) risk screening and assessment, (4) develop a single CC care plan, (5) deliver CC, and (6) review and improve CC delivery. These essential elements are closely interconnected and cover various stages or processes that a patient may experience in care delivery. They are also relevant to the well-established nursing process, encompassing assessment, diagnosis, planning, implementation, and evaluation, while also incorporating specific aspects such as identifying patients’ needs and preferences and minimising the risk of harm. As per Xiong et al.‘s review [12], risk screening and assessment and minimising patient harm is a unique component of the Australian national standard for CC. This review identified a significant knowledge gap regarding the impacts of addressing specific harms in a national standard for CC and emphasised the need for further studies to delve into this issue.

To support the implementation of the CCS, the ACSQHC also developed a conceptual model (referred to as “ACSQHC model”) (ACSQHC 2018). This model describes the key elements that health service organisations need to consider when implementing the CCS. The ACSQHC model also serves as a tool for identifying areas of improvement, enabling organisations to address gaps and enhance the delivery of CC. According to this model, the cultural conditions and systems and processes for CC fall into three groups: “1. a focus on patient experience, 2. systems, processes and protocols to deliver CC, and 3. organisational culture and governance that supports a CC approach” (p. 9) [13]. A focus on patient experiences is at the top of this pyramid-shaped model, emphasising the importance of person-centred principles in the policies, processes, and governance of the organisation for implementing the CCS.

Although the implementation of the CCS in hospitals is mandated, achieving 100% compliance has proven to be challenging, and the degree of compliance varies [14]. Two years after the CCS came into effect, 15% of the assessed health service organisations did not meet all the requirements of the CCS and 12% were provided with recommendations to meet the actions. This indicates underperformance of the implementation of the CCS [14]. In 2021, the ACSQHC surveyed health service organisations that had undergone accreditation assessments to identify the challenges associated with implementing the four criteria of the CCS and suggestions for resources [15]. This survey revealed that the most challenging criteria was developing a CC plan due to reasons such as no standard care plan used by all disciplines and difficulties in care planning with multidisciplinary teams (MDTs).

The ACSQHC survey primarily focused on investigating the implementation challenges of the CCS but did not explore potential facilitators. It targeted the contact person of health service organisations that are typically responsible for accreditation, which may not have captured the perspectives of individuals working directly with patients. Additionally, there is a notable lack of studies examining the impacts of the CCS. Therefore, there remains a research gap in understanding the implementation challenges, potential facilitators, and impacts of a national standard for CC [12]. A lack of knowledge of the implementation and impacts of a new care standard may hinder the sustainability of the care standard initiatives, waste financial resources, energy and time and increase the costs imposed on patients and governments [16]. The aim of our study is to develop a national picture of care professionals’ self-reported knowledge, experiences, and perceptions about the implementation and impacts of the CCS in Australian acute care hospitals. The findings of our study will contribute to a better understanding of the implementation and impacts of the Australian CCS in acute care hospitals and provide recommendations for further improvement. Moreover, these insights have the potential to advance the implementation of a national standard for CC in acute care hospitals not only within Australia but also internationally, benefiting healthcare systems worldwide.

## Method

### Study design

The present study is part of a larger project exploring the implementation and impacts of the national standard for CC in Australian acute care hospitals from the perception of care professionals, patients, and informal carers [17]. This study uses survey methodology to understand the care professional perspective in a hospital setting, specifically acute care. A cross-sectional survey was created, distributed, and administered using the Checkbox survey platform (Checkbox Technology, Inc.), and was conducted from October 1, 2022, to April 30, 2023. This study is reported in compliance with the Consensus-Based Checklist for Reporting of Survey Studies [18].

### Questionnaire development

As no existing survey instruments were identified through our previous literature review, a questionnaire was developed for this study, underpinned by the Commission’s evaluation of the CCS survey [15], the ACSQHC model [13], and previous literature [6, 12]. BX, MMK, and CS participated in a collaborative and iterative process in the early stages of questionnaire development, refining and pre-testing the questions to ensure content validity and appropriate scope.

To pilot test the survey, we attached five survey evaluation questions (Supplementary Table 1) to the questionnaire and distributed it to 60 potential participants working at Australian acute care hospitals from July 1 to August 31, 2022. Participants were asked to fill in the questionnaire and then review the five aspects (i.e., readability, adequacy, relevance, practicality, and ethicality) of the survey. Twenty-nine care professionals responded to the online questionnaire, 21 met the inclusion criteria (care professionals who worked in acute care hospitals in Australia and had heard about the CCS) and 12 (*n* = 12/29, 41%) completed the validity questions.

Based on the results of the pilot survey (Supplementary Table 2), items, wording, and question order were revised before the subsequent, larger, formal investigation. Considering not all care professionals working in Australian hospitals knew about the CCS, we revised the screening questions and wording of the survey introduction to exclude these potential participants. Because of the importance of the CC plan in CC delivery, we added a question about the CC plan. Based on the results of the pilot survey, we also revised the response categories of two demographic questions. Due to incomplete responses, we moved the demographics section from the beginning to the end of the survey. Because the survey was anonymous, we could not send the revised questionnaire to the original respondents of the pilot test for feedback. Additionally, due to time and resource constraints, the revised version was reviewed by our research team for feedback before the formal launch.

### Final questionnaire

The final online questionnaire consists of six sections, including: (1) screening section to confirm eligibility; (2) demographic section to obtain information regarding gender, employment location, rurality of location [19], type of hospital, work area, profession, leadership, and working experiences; (3) knowledge section to assess perceived knowledge about the CCS and confidence in performing CC; (4) practice section to examine received support for the CCS implementation and practices in CC delivery; (5) barriers and facilitators section to identify factors associated with the effective implementation of the CCS; and (6) perceived effects section to evaluate the perceived effects of the CCS implementation on patient care and health outcomes.

The questionnaire examines perceptions of 15 common effects of CC, as indicated by the review paper [6], and the definitions of these effects were included in the questionnaire (Supplementary Table 3).

The questionnaire includes 15 multiple-choice questions (including three matrix questions) and five free-text questions. The final version of the survey can be found in Supplementary Tables 3 and Supplementary Questionnaire.

### Population and sampling

The population of interest is care professionals (doctors, nurses, and allied health professionals) who worked in acute care hospitals in Australia and had heard about the CCS at the time of the survey.

We employed convenience and snowballing sampling techniques to distribute the survey through our research team and organisation, healthcare organisations and facilities, and clinical networks using various methods, including websites, newsletters, magazines, emails, and social media platforms (Twitter, Linkedin, Facebook, and Instagram). In the survey invitation, recipients were informed that they were welcome to share the survey with their networks.

### Sample size

According to Roscoe’s Simple Rule of Thumb, samples of 30 or more are recommended for one sub-sample [20]. The maximum number of sub-groups based on our demographic question is 8. According to Weisberg & Bowen [21], a sample size of 400 respondents is required if an error level of 5% is accepted in an e-survey. A sample size of at least 400 meets both criteria and was the minimum goal for our study.

### Consent

Completion of the survey implied consent. Participation in the study was voluntary and confidentiality was assured as no identifiable information was collected. Participants were allowed to skip questions, except for the questions related to participant eligibility. Ethical approval was granted by the University of Queensland Human Research Ethics Committee (ID: 2022 /HE001036).

### Data management

Survey data was collected without identifiable information and stored on the University of Queensland’s secure Research Data Management system, with access provided only to the researchers directly involved in its analysis.

### Quality control

Five questions in the effect section examine negative outcomes, while the rest investigate positive outcomes. Respondents who chose “increase” or “decrease” for all the outcomes were deemed invalid and were excluded (reported as ”not responding logically”). Respondents who had the same IP address and had the same string of five or more words in the open-ended questions were considered repetitious and only their most recently completed responses were included in the analysis.

### Statistical analysis

Survey data were included if at least one survey question related to the CCS implementation or CC delivery was answered. As the number of respondents completing each question varied, proportions reported were based on valid responses to each question.

Quantitative data were collated in Microsoft Excel for Mac (version 16.50) with statistical analyses carried out using R (version 4.1.0) and RStudio (version 1.4.1717) software. A two-sided *p* value of less than 0.05 was used to indicate statistical significance. Quantitative data were presented using descriptive statistics, including mean, standard deviation, median, range, cross-tabulations and proportions. Because all the variables were not normally distributed, we primarily used the median to provide a more accurate representation of the central tendency for non-normally distributed data. When the median values were the same, we reported the mean to highlight the differences.

Kruskal-Wallis tests were employed to assess potential differences in the variables within the ‘knowledge’ and ‘support’ sections among various demographic groups, as the assumption of normality was violated in their distributions. Post hoc analyses for Kruskal-Wallis were performed using Dunn’s test adjusted with the Bonferroni method. Chi-square tests were used to explore potential differences in the variables within the ‘effect’ section across demographic groups concerning organisation, profession, and leadership. Fisher’s exact tests were conducted due to the violation of the assumption of expected observations exceeding 5 within demographic groups for gender, location, work unit, and work experience. Post hoc analyses for both Chi-square and Fisher’s tests were adjusted using the Bonferroni method.

Free-text responses were analysed with NVivo software (version 12.3.0), coding thematically using a deductive approach [22, 23]. During the analysis, BX began by thoroughly reading through the free-text responses to grasp the content and context. As BX read, BX started to notice recurring patterns, ideas, and concepts. These emergent patterns were then assigned codes, which served as labels for these common elements in the data. As the coding progressed, BX grouped similar codes together to form initial themes. Subsequently, all authors reviewed the themes.

## Results

### Demographics

Our online survey received 864 responses, among which 215 responses were excluded due to: not working in an acute care hospital (*n* = 20) or having not heard about CCS (*n* = 99) or both (*n* = 9); starting the survey but not proceeding to the main body of the survey (*n* = 32); not responding logically (*n* = 8) or repeating responses (*n* = 65). After removing those invalid or ineligible responses, 649 responses were included in the analysis. The median time for completing the survey was 7 min (Q1 = 4 min, Q3 = 12 min).

Our sample consisted of registered nurses or midwives (44%, *n* = 180), allied health professionals (31%, *n* = 130), and doctors (25%, *n* = 104) and 40% (*n* = 164) were a manager/director/leader in their profession. Our sample mirrors the distribution in the Australian health workforce (*n* = 642,000), which consists of 55% nurses (*n* = 350,000), 29% allied health professionals (*n* = 187,500), and 16% doctors (*n* = 105,300) [24]. Table 1 displays the characteristics of our sample. Females made up 52% (*n* = 216) of the sample, with 3–10 years of working experience comprising 58% (*n* = 240). All Australian states and territories were represented in the study, with one-third of respondents working in Queensland (35%, *n* = 146). Additionally, 48% (*n* = 201) of the respondents were from regional areas, and three-quarters worked in public hospitals (76%, *n* = 316). 47% (*n* = 188) of the respondents were from the emergency department (ED) or general medicine.

**Table 1:** Characteristics of the sample

Characteristics	Count (%)
Gender (*n* = 416)	
Female	216 (51.9)
Male	190 (45.7)
Unspecified	10 (2.4)
State and territory (*n* = 416)	
Queensland	146 (35.1)
South Australia	66 (15.9)
Australian Capital Territory	56 (13.5)
New South Wales	51 (12.3)
Victoria	35 (8.4)
Tasmania	22 (5.3)
Northern Territory	22 (5.3)
Western Australia	18 (4.3)
Location (*n* = 415)	
Metro	156 (37.6)
Regional	201 (48.4)
Rural	47 (11.3)
Remote	11 (2.7)
Organisation (*n* = 416)	
Public	316 (76.0)
Private	100 (24.0)
Work area/unit (*n* = 404)	
Emergency department	108 (26.7)
General Medicine	80 (19.8)
ICU	59 (14.6)
Surgery	55 (13.6)
Other	102 (25.2)
Profession (*n* = 414)	
Registered nurse/midwife	180 (43.5)
Medical doctor	104 (25.1)
Pharmacist	67 (16.2)
Other allied health professional	63 (15.2)
Being a manager/director/leader in their profession (*n* = 414)	164 (39.6)
Work experiences (*n* = 414)	
Less than 3 years	51 (12.3)
3–10 years	240 (57.9)
11–20 years	68 (16.4)
More than 20 years	55 (13.3)

Note: The number of respondents completing each question varied, leading to variations in the number of responses. Location is classified according to the Modified Monash Model. Available from https://www.health.gov.au/topics/rural-health-workforce/classifications/mmm

Doctors had a higher proportion of males (72%, *n* = 75) than nurses (27%, *n* = 48) in the questionnaire respondents. Nurses (71%, *n* = 39) had a higher proportion of over 20 years of work experience than doctors (13%, *n* = 7). Public acute care hospitals had a higher proportion of nurses (47%, *n* = 147) and leaders (42%, *n* = 134) among the questionnaire respondents compared to private acute care hospitals (33%, *n* = 33; 30%, *n* = 30).

The results from the knowledge, practice, and perceived effects sections are presented below. The results from the barriers and facilitators section are presented in another paper.

### Perceived knowledge

The CCS is mandated in all acute care hospitals in Australia and its implementation relies on joint efforts from all care professionals. A 6-point Likert scale (0 = none to 5 = very high) measured the self-assessment of care professionals’ knowledge of the CCS. Analysis showed that on average respondents self-reported a ‘moderate’ level of knowledge of the CCS (median = 3/5) (Table 2).

The median level of self-reported knowledge for males (median = 4/5) was significantly higher than for females (median = 3/5) with *z* = 3.4 and an adjusted *p* value of 0.002. Respondents from private acute care hospitals (median = 4/5) were identified as having a significantly higher average level of self-reported knowledge than those from public acute care hospitals (median = 3/5, χ^2^(1) = 6.3, *p* = 0.012). On average, medical doctors (median = 4/5) sel-reported a higher level of knowledge than registered nurses (median = 3/5, *z* = 3.0, *p*_*adj*_=0.008). On average, respondents from the Australian Capital Territory self-reported a significantly higher level of knowledge (median of 4/5 vs. 3/5) than those from New South Wales (*z* = 4.7, *p*_*adj*_<0.001), Queensland (*z* = 4.2, *p*_*adj*_<0.001), and Victoria (*z* = 5.6, *p*_*adj*_<0.001). No differences in the average level of self-reported knowledge were found in the rurality of locations, work units, working experiences, and having a leadership role or not (*p* > 0.05).

### Perceived confidence

Performing CC involves six essential elements, and respondents were required to rate their confidence in performing them using a 5-point Likert scale (1 = very low to 5 = very high). On average, respondents self-reported a ‘high’ level of confidence in performing each of the six elements of CC (median = 4/5). However, the lowest average level of perceived confidence was observed in developing a single CC plan (mean = 3.53, SD = 1.07) (Supplementary Fig. 1, Table 2).

On average, doctors (mean = 4.04, SD = 0.80) and nurses (mean = 3.82, SD = 0.84) self-reported a higher level of confidence in clinical assessment and diagnosis than allied health professionals (mean = 3.58, SD = 9.52), with doctors being significantly more confident (*z* = 3.8, *p*_*adj*_<0.001). Care professionals with 11–20 years of work experience (mean = 4.11, SD = 0.77) self-reported higher confidence in clinical assessment and diagnosis compared to those with more than 20 years of work experience (mean = 3.90, SD = 0.87, *z* = 1.2, *p*_*adj*_=1.00), those with 3–10 years of work experience (mean = 3.72, SD = 0.88, *z* = 3.2, *p*_*adj*_=0.009) and those with less than 3 years of work experience (mean = 3.68, SD = 0.89, *z* = 2.6, *p*_*adj*_=0.052). No statistically significant differences in the average level of perceived confidence were found in other elements of CC among different demographic groups (*p* > 0.05).

### Perceived support

According to the ACSQHC model, hospitals are required to provide organisational support to facilitate the implementation of the CCS. The perceived organisational support was measured using a 5-point Likert scale (1 = very low to 5 = very high). On average, respondents self-reported receiving a ‘moderate’ level (median = 3/5) of organisational support regarding education and training, systems and processes that support CC, and equipment and tools to implement the CCS (Supplementary Fig. 2, Table 2). In contrast, respondents self-reported receiving an average of ‘high’ level (median = 4/5) of support in areas such as leadership across the organisation, ongoing quality improvement, and standardisation of hospital practices and policy. Perceptions towards support in education and training were at the lowest average level among the six aspects (mean = 3.37, SD = 1.03).

Among the care professionals surveyed, doctors self-reported receiving significantly more support than nurses in leadership across their organisations (*z* = 2.5, *p*_*adj*_=0.034) and ongoing quality improvement (*z* = 3.0, *p*_*adj*_=0.009).

When it came to support in education and training, doctors also self-reported receiving significantly more support than nurses (*z* = 3.7, *p*_*adj*_<0.001) and allied health professionals (*z* = 2.6, *p*_*adj*_=0.028). Males self-reported receiving significantly more support than females (*z* = 3.0, *p*_*adj*_ = 0.008), care professionals from private acute care hospitals self-reported receiving significantly more support than those in public acute care hospitals (χ^2^(1) = 4.2, *p* = 0.040), and care professionals with 3–10 years of work experience self-reported receiving significantly more support than those with over 20 years (*z* = 3.0, *p*_*adj*_=0.018).

In terms of support for equipment and tools, the findings indicated that doctors also self-reported receiving significantly higher levels of support compared to nurses (*z* = 2.9, *p*_*adj*_=0.011). Males self-reported receiving significantly more support than females (*z* = 3.2, *p*_*adj*_=0.004), care professionals in private acute care settings self-reported receiving significantly more support than those in public acute care hospitals (χ^2^(1) = 7.1, *p* = 0.008), and care professionals with 3–10 years of experience self-reported receiving significantly more support than those with over 20 years (*z* = 3.1, *p*_*adj*_=0.011).

Regarding support for systems and processes supporting CC, allied health professionals self-reported receiving more support in this aspect than nurses (*z* = 2.8, *p*_*adj*_=0.015). Care professionals in private acute care hospitals or working in intensive care units self-reported receiving more support than those in public acute care hospitals (χ^2^(1) = 11.5, *p* < 0.001) or working in general medicine (*z* = 3.3, *p*_*adj*_=0.011). Additionally, care professionals with less than 20 years of work experience self-reported receiving more support than those with over 20 years (less than 3 years: *z* = 2.9, *p*_*adj*_ =0.020; 3–10 years: *z* = 3.6, *p*_*adj*_ =0.002, 11–20 years: *z* = 3.4, *p*_*adj*_=0.004).

Support in standardisation of hospital practices and policy revealed that doctors self-reported receiving more support compared to nurses (*p*_*adj*_=0.038). Males self-reported receiving more support than females (*z* = 2.8, *p*_*adj*_=0.017), and professionals in private acute care hospitals self-reported receiving more support than those in public acute care hospitals (*p*_*adj*_=0.037). Furthermore, professionals with 3–20 years of experience self-reported receiving more support than those with over 20 years (3–10 years: *z* = 3.1, *p*_*adj*_=0.012, *z* = 3.0, 11–20 years: *p*_*adj*_=0.018).

**Table 2:** Self assessment of knowledge about the comprehensive care standard, confidence in performing comprehensive care, and organisational support in implementing the comprehensive care standard

Items	*n*	None	Very low	Low	Moderate	High	Very high	Median	Mean	SD
		0	1	2	3	4	5			
**Knowledge of the CCS**	*636*	8 (1.3)	24 (3.8)	103 (16.2)	189 (29.7)	236 (37.1)	76 (11.9)	*3*	*3.33*	*1.08*
**Confidence in performing CC**										
1. *Clinical assessment and diagnosis*	637		11 (1.7)	37 (5.8)	193 (30.3)	273 (42.9)	123 (19.3)	4	3.72	0.90
2. *Identify goals of care*	636		9 (1.4)	45 (7.1)	177 (27.8)	254 (39.9)	151 (23.7)	4	3.78	0.94
3. *Risk screening and assessment*	635		10 (1.6)	41 (6.5)	216 (34.0)	234 (36.9)	134 (21.1)	4	3.69	0.93
4. *Develop a single CC plan*	639		27 (4.2)	80 (12.5)	185 (29.0)	222 (34.7)	125 (19.6)	4	3.53	1.07
5. *Deliver CC*	636		17 (2.7)	54 (8.5)	174 (27.4)	251 (39.4)	140 (22.0)	4	3.70	0.99
6. *Review and improve CC delivery*	628		23 (3.7)	50 (8.0)	188 (29.9)	228 (36.3)	139 (22.1)	4	3.65	1.03
**Organisational support for CCS implementation**										
1. *Leadership across the organisation*	557		14 (2.5)	53 (9.5)	175 (31.4)	232 (41.7)	83 (14.9)	4	3.57	0.94
2. *Education and training*	556		25 (4.5)	80 (14.4)	189 (33.9)	186 (33.5)	76 (13.7)	3	3.37	1.03
3. *Equipment and tools*	555		18 (3.2)	75 (13.5)	188 (33.9)	178 (32.1)	96 (17.3)	3	3.47	1.03
4. *System and process that support CC*	553		23 (4.2)	74 (13.4)	187 (33.8)	179 (32.4)	90 (16.3)	3	3.43	1.04
5. *Standardisation of hospital practices and policy*	555		20 (3.6)	62 (11.2)	191 (34.4)	201 (36.2)	81 (14.6)	4	3.47	0.99
6. *Ongoing quality improvement*	549		22 (4.0)	68 (12.4)	170 (30.9)	199 (36.2)	90 (16.4)	4	3.49	1.03

Note. CCS: Comprehensive Care Standard, CC: comprehensive care

The number of respondents completing each question varied, leading to variations in the number of responses

### Consumer involvement

According to the ACSQHC model, the implementation of the CCS should focus on patient experiences, and involving consumers is essential to reflect the person-centred principle in the policies, processes, and governance of the organisation. Participants were asked whether their organisations formally involved patients or care partners (also known as consumers) in the preparation, training, or implementation process of the CCS. Of the 449 respondents, 44% (*n* = 199) of respondents reported “Yes” to this question, 22% (*n* = 150) reported “No”, while 33% (*n* = 100) were unsure if their organisations involved consumers in these processes.

124 respondents reported the approaches to consumer involvement in their organisation. Seven common themes were identified and were presented here with examples (Table 3).

Establishing a committee or advisory group consisting of consumers was a common approach described that allowed consumers to provide input and perspectives on the implementation of the CCS. Another common approach was including consumer representatives in various working groups and meetings related to the CCS, ensuring that their voices were heard and considered in decision-making processes. Collaborating with consumers to jointly design and develop work processes and programs related to CC, and incorporating their perspectives and preferences were also mentioned by some respondents. Some respondents reported that their hospital offered educational resources and training programs specifically tailored for consumers to increase their understanding of CC principles and their role in their care. Actively involving consumers in making decisions about their own care, encouraging their participation, and supporting their autonomy were identified as routine practices. Additionally, some respondents reported that their hospital involved patients in sharing their stories and experiences related to CC, which increased publicity and awareness, promoted a patient-centred care culture, and fostered empathy among healthcare providers. Furthermore, respondents highlighted the importance of actively seeking feedback from consumers regarding their experiences of care, listening to their suggestions and concerns in various ways, and taking appropriate actions based on their feedback.

**Table 3:** Approaches acute care hospitals used to involve consumers in the preparation, training, or implementation process of the comprehensive care standard

Theme	Exemplar quotes
1. Consumers committee or advisory group	“There is a partnering with consumers committee that reviewed the implementation of comprehensive care - risk screening and care planning tools.” (Nurse, ACT) “Patient and Family Advisory Councils (PFACs) … PFACs provide feedback and input on policies, procedures, and programs.” (Doctor, VIC)
2. Consumer representatives in working groups and meetings	“Consumer representation on Standard 5 committee and some sub working groups under standard 5 - eg falls.” (Nurse, QLD)
3. Codesign with consumers on work processes and programs	“There have been bodies of work undertaken which have involved a co-design process, including work around improving mealtimes and mealtime environments, and falls. … Consumers have been regularly involved in the development of risk screening and care planning processes.” (Allied health professional, QLD)
4. Providing consumer with education and training on comprehensive care	“Several routines, talk session have been set up to educate patients and care partners on comprehensive care standards.” and “Provide patients with critical health information and education and engage them in joint decision-making.” (Nurse, NSW) “Patients actively partook in programs organised by my organisation in training for standardised comprehensive care.” (Allied health professional, SA)
5. Engaging consumers in their own care	“Discuss treatment aims and care plan with patient. … carers consulted when required for planning.” (Nurse, NSW) “Talk to the patient about the importance of comprehensive care and involve the patient in the discussion.” (Doctor, SA)
6. Involving consumers in storytelling and experience sharing	“Consumers talk at workshops, provide feedback” (Unknown) “Patient stories/experiences” (Nurse, NSW) “Constantly summarise experience in the work, timely promote in the whole hospital, so as to benefit more patients.” (Nurse, ACT)
7. Seeking feedback from consumers and acting on it	“Opinions were sampled from patients and care partners through survey, and a kind of service box.” (Doctor, QLD) “… That feedback will be considered in developing the updated version of the paper CCP (comprehensive care plan) tool.” (Allied health professional, TAS)

Note. NSW: New South Wales, VIC: Victoria, QLD: Queensland, SA: South Australia, TAS: Tasmania, ACT: Australian Capital Territory

### Care plan

Developing a single CC plan is an essential element of CC. Participants were asked about the proportion of patients who had a care plan that met the requirement of the CCS (referred to as “CC plan”) in their unit, and the response was given on a 5-point Likert scale with options ranging from “none” (1) to “all” (5). 4% (*n* = 17) of respondents reported that “all” patients in their unit had a CC plan. Respectively, 29% (*n* = 120), 28% (*n* = 116), and 21% (*n* = 86) reported “most”, “half”, and “some” of the patients had a CC plan. On the other hand, 18% (*n* = 74) of respondents reported that “none” of the patients in their unit had a CC plan.

69 respondents reported the reasons for not all having a CC plan. Seven themes of not providing a CC plan were identified and were presented here with examples (Table 4).

Some respondents believed that non-compliant care plans with the CCS requirement were attributed to factors resulting from individuals, such as a lack of knowledge about creating a CC plan, insufficient motivation and commitment, and reliance on nursing staff and a lack of teamwork. On the other hand, others identified challenges related to the hospital itself such as scarcity of resources, including equipment, tools, staffing, and funding. This scarcity of resources led to competing priorities between clinical care and documentation. The challenges of documentation and limitations within the system and processes exacerbated the situation. Additionally, some respondents pointed out that the environment or setting, especially in the ED and day procedure environment, posed challenges for achieving compliant care plans.

**Table 4:** Factors contributing to non-compliant care plans with the requirements of the comprehensive care standard

Theme	Exemplar quotes
Lack of knowledge and awareness	“Lack of knowledge” (Nurse, NSW) “I don’t even know what should be included to meet the standard” (Doctor, QLD) “I have not seen a care plan perse. … I look at the parts of the chart that pertain to my care area. …there might be care plans but I’ve not seen them” (Allied health professional, QLD)
Lack of motivation and commitment	“Not activated by all staff” (Nurse, QLD) “No MDT [multidisciplinary team] buy-in” (Nurse, NSW) “Under recognition of the Standard and underuse of formal care plans in my clinical area (emergency medicine)” (Doctor, QLD)
Nursing dependent and lack of teamwork	“Nursing dependent” (Nurse, NSW) “While it was presented as a document that all medical, nursing and allied health staff could contribute to, in reality, the onus is on nursing staff to complete the entire document.” (Nurse, TAS)
Lack of resources	“Hospitals don’t have the money to fill nursing positions.” (Unknown) “lack of workforce / nurse ratio breeches due to workforce shortages due to covid” (Allied health professional, VIC) “Using old health equipment for operation” (Allied health professional, NSW) “Time constraints, staff shortage and competing priorities ie clinical care vs documentation.” (Nurse, NSW) “Time constraints, keeping up with changes to plans…” (Allied health professional, QLD)
System and process limitations	“Appropriate preparation for the comprehensive care process has not been made.” (Nurse, QLD) “Then there is system and process barriers e.g to update an electronic form it takes two years to have the system upgrade with the service provider” (Nurse, QLD)
Documentation challenges	“It is difficult to complete documentation to comply with the requirements due to the volumes of patients and ieMR (Integrated Electronic Medical Records) functions.” (Nurse, QLD) “We are currently using a paper based plan that is not suited to our needs. No one wants to fill it in. most of the patients care needs are already evident on our electronic system.” (Nurse, NSW) “The new Comprehensive Care Plan that was released is an approximately 36-page admission document that is overwhelming and excessive. It rarely gets completed in full due to the length and complexity of it.” (Nurse, TAS)
Environment or setting	“Busy emergency department” (Nurse, NSW) “In ED [emergency department] we do more of an action plan then a comprehensive care plan for majority of pts [patients] simply due to their reason from presenting” (Nurse, QLD) “We are a day procedure environment so care plan is not formally written for these patients” (Nurse, QLD) “We do not have good care plans for outpatients and people who attend frequently for chronic health conditions. Our inpatients do have daily care plans…” (Nurse, QLD)

Note. NSW: New South Wales, VIC: Victoria, QLD: Queensland, TAS: Tasmania

### Perceived effects

**Fig. 1:**
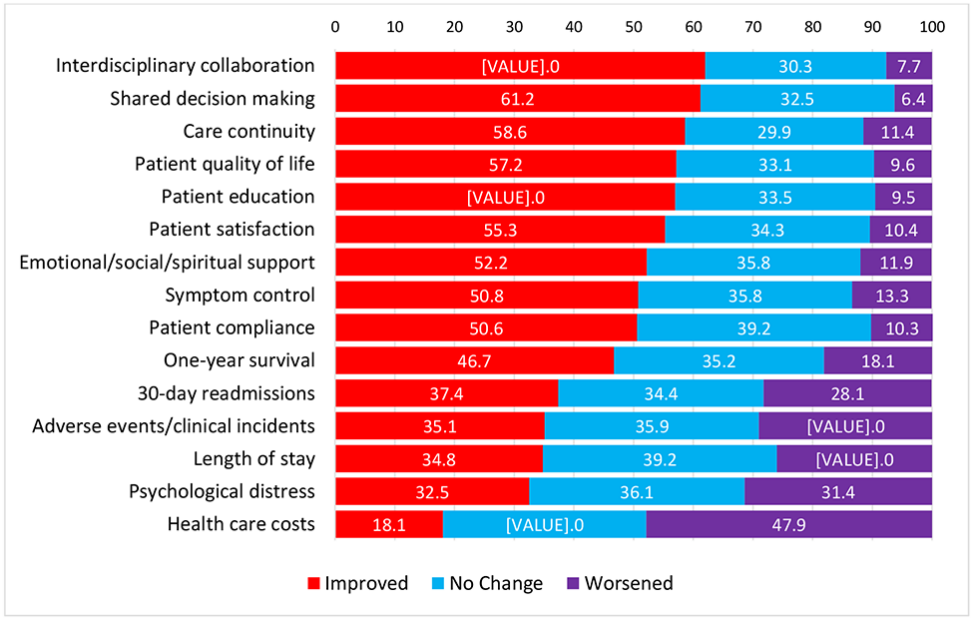
Effects of the introduction of the Comprehensive Care Standard on patient care and health outcomes ranked in the descending order of improved effects

The questionnaire examined 15 effects that are commonly examined for evaluating CC. A 3-point rating scale (“worsened”, “no change”, “improved”) measured the perceived effects of the CCS. A small proportion of participants were not aware of the listed effects, ranging from 5% (shared decision-making and Interdisciplinary collaboration) to 20% (one-year survival) (Supplementary Table 4). Among those who were aware, about one-third (ranging from 26 to 35%) of the participants thought there were no changes in the effects of the introduction of CCS on patient care and health outcomes (Fig. 1).

Among those who were aware, more than half of the respondents thought there were improvements in interdisciplinary collaboration (62%, *n* = 233), shared decision-making (61%, *n* = 230), care continuity (59%, *n* = 200), patient quality of life (57%, *n* = 190), patient education (57%, *n* = 209), patient satisfaction (55%, *n* = 192), and emotional/social/spiritual support (52%, *n* = 188), symptom control (51%, *n* = 183), and patient compliance (51%, *n* = 182) after the introduction of the CCS.

More than one-fourth of respondents believed there were worsened outcomes in length of stay (26%, *n* = 88), 30-day readmission (28%, *n* = 94), adverse events/clinical incidents (29%, *n* = 104), and psychological distress (31%, *n* = 112). An important caveat to these findings is that a greater proportion of participants thought that these metrics had improved since the introduction of the CCS, however the proportions thinking these had improved was towards the lower end of the Fig. 1, below.

With all of these effects, a greater proportion of respondents did feel that patient care and health outcomes had improved, with the exception of healthcare costs. A greater proportion of respondents believed that health care costs increased (48%, *n* = 159) than those who thought they decreased (18%, *n* = 60) due to the introduction of the CCS.

Nurses, females, with more than 20 years of work experience, having leadership roles, and working in public acute care hospitals, tended to report ‘no change’ more frequently than ‘improved’ for certain outcomes compared to doctors, males, with less than 20 years of work experience, not having leadership roles, and working in private acute care hospitals. In metropolitan areas, there was a tendency for some outcomes to ‘improve’ or remain ‘unchanged’ compared to ‘worsening’, and ‘improvement’ was more common than ‘no change’ or ‘worsening’ for certain outcomes, in contrast to regional or remote/rural areas. Departments such as general medicine and others showed a tendency for ‘improved’ or ‘unchanged’ outcomes compared to ‘worsening’ for certain outcomes, as opposed to departments like ICU, ED, or surgery. Details of these specific outcomes by demographics are provided in the Supplementary Table 5.

## Discussion

The aim of our study was to develop a national picture of care professionals’ self-reported knowledge, experiences, and perceptions about the implementation and impacts of the NSQHS CCS. In the five years since the release of the CCS, care professionals self-reported having a moderate level of knowledge of the CCS, a high level of confidence in performing CC, but experiencing only a moderate level of organisational support. This study also revealed seven common approaches for involving consumers in the CCS implementation process and highlighted seven recurring themes that contribute to non-compliant care plans aligned with CCS requirements. From the care professional perspective, many positive changes from the introduction of the CCS on patient care and health outcomes had been observed. More support in education and training as well as resources to support the development of the CC plan are needed to support the implementation of the CCS. Findings provide insights on what can be done to further improve the implementation of the CCS in Australia and might advance the implementation of national standards for CC internationally.

Despite the CCS being a mandatory national standard and its implementation relying on joint efforts from all care professionals, their perceived understanding of the CCS remains relatively limited. The lack of knowledge may result from ineffective communication strategies about the CCS and insufficient training and education. Previous research has highlighted the importance of communication in the delivery and dissemination of a new clinical policy within or beyond hospitals, thus facilitating more effective implementation [25]. However, it remains unclear how the CCS was communicated within and beyond the organisation [12]. Future research in this area would help the ACSQHC and hospitals identify strategies to improve the communication of the CCS, thus improving care professionals’ “awareness-knowledge” [25] of this Standard. Surprisingly, we found that males, doctors, and care professionals from private acute care hospitals believed they had higher levels of perceived knowledge of the CCS than females, nurses, and care professionals from public acute care hospitals. The differences in gender and hospital type may be attributed to variations in the professions. Based on our survey data, the workload of meeting the requirements of the CCS was primarily placed on nurses, despite their lower level of knowledge of the CCS compared to doctors. Nurses were frequently instructed on how to perform tasks without being provided with the underlying rationale. This highlights the importance of enhancing nurses’ knowledge of the CCS, as greater knowledge of guidelines and recommendations can increase nurses’ adherence to and compliance with the principles of CC [26].

Although respondents’ knowledge level of CCS was moderate, their confidence level in their ability to perform CC was high. This may be because many clinicians had received training in various aspects of CC before the introduction of the CCS (such as risk screening and multidisciplinary teamwork). However, the lowest level of confidence in performing CC was in developing a CC plan, which parallels previous findings that developing a CC plan was the most challenging criterion to implement and an area for improvement [14, 15]. The lack of confidence in developing a CC plan corresponds to the low compliance of care plans with the CCS requirements. This low compliance is consistent with previous research findings that a patient-centred care plan is often not visible in the patient record [27]. Addressing the challenges of CC plan implementation requires a multifaceted approach that targets both individual and systemic factors. At the individual level, targeted training to enhance staff knowledge, incentives to boost motivation and commitment, and inter-professional collaboration initiatives to enhance teamwork and reduce reliance on nursing staff are essential. On a systemic level, hospitals must address resource scarcity, streamline documentation processes, and re-evaluate and restructure systems and processes to overcome these limitations.

The development of a CC plan should be a collaborative effort involving the MDTs rather than the sole responsibility of nursing staff. Research indicates multidisciplinary collaboration in care planning is essential for achieving better patient outcomes [28]. However, the current practice of paper-based documentation poses significant challenges for MDTs, as it complicates the process of generating and updating across disciplines and settings. This approach often leads to redundant and time-consuming tasks of recording repetitive documents, which does not add value to actual patient care [29]. Fortunately, the shift towards electronic medical records (EMR) offers a promising solution, enabling intelligent and efficient documentation and facilitating better collaboration. However, most existing EMR programs have focused on the development of care plan applications for use by a single discipline (e.g., nursing) or department [30]. This silo approach is unlikely to meet the intended effect of the care plan that improves MDT communication and does not reflect an MDT-based approach to care planning and delivery. This highlights a need to build Integrated Electronic Medical Records (ieMR) that supports the integration of care planning documentation into workflows that exist across the continuum and facilitate input by all team members (including patients) to truly reflect an MDT approach [31, 32]. However, implementing ieMRs presents its own challenges, especially if these systems fail to meet the needs of care professionals, particularly in terms of functionality [33]. It is critical that ieMRs are designed to support effective communication and coordination, rather than simply serving as a tick-box task. Actual patient care should always be emphasised despite changes in policies and clinical administrative processes [27, 29]. By addressing the systemic barriers to effective care planning, hospitals can better support their staff and improve patient care.

Supported by both structured questions and free-text responses, respondents indicated a clear desire for increased knowledge and training, as well as for more resources and improved systems and processes, especially from nurses. Lack of education and training may hinder knowledge, awareness, and belief in implementing the CCS. Care professionals require education, training, and developmental support to grasp CC principles and how various components contribute to its delivery, particularly within their specific settings [13]. Training should cover the adoption of new policies, processes, approaches, and tools throughout different organisational layers, promoting alignment with the goal of delivering CC for a more consistent approach [13]. Lack of resources (such as funding, staffing, and equipment) may have greatly impacted the implementation of the CCS. In line with previous research, high workload, poor staffing, and time pressure are very common barriers to adherence to compliance with guidelines and recommendations, especially in low-resource settings and at the time of COVID-19 [34, 35]. Previous research reveals the availability of resources, equipment and tools, and digitalisation increased the likelihood of adherence to patient safety principles [26]. Furthermore, the establishment of systems and processes to support the delivery of CC [13], ensuring standardised content, information, messaging, and terminology models for care plans across various disciplines and settings, is crucial for effective communication and seamless continuity of care. Achieving this high level of standardisation necessitates coordination at both local and national levels.

Providing care that responds to consumers’ needs is a requirement of the NSQHS Standards [36], as part of the CCS and the Partnering with Consumers Standard [11]. This responds to the growing consensus that involving consumer in the development, implementation and evaluation of healthcare contributes to more targeted initiatives, better resource utilisation, and improvement in the safety, quality, and overall performance of health services organisations [37, 38]. Evidence suggests that patient involvement in goal setting and ongoing status updates is also crucial because patients who actively engage in their disease management are less likely to be re-hospitalised after an acute exacerbation [39]. This underscores the importance of integrating consumer engagement strategies into clinical workflows, not merely as a compliance measure but as a core component of effective healthcare delivery. Our study identified seven common approaches to consumer involvement. A one-size-fits-all approach to consumer partnership is neither feasible nor desirable. Instead, strategies should be tailed to fit the specific nature and context of each organisation, ensuring that consumer engagement is meaningful and sustainable.

Our research findings hold significant implications for healthcare practice and policy. Following the introduction of the CCS, our study indicates that positive perceived changes were observed in patient care and health outcomes. This aligns with the existing body of literature, exemplified by a review of 16 papers on the effectiveness of CC conducted by Grimmer et al. [6]. However, it’s noteworthy that care professionals in our study did not uniformly perceive significant reductions in adverse events at this time. These varied perspectives shed light on the complexity of the Australian national standard for CC, which includes a unique emphasis on minimising patient harm, and underscores the necessity for further examination and refinement in this area to enhance patient safety and healthcare quality. Contrary to the findings of Grimmer et al. [6], care professionals in our study showed a perception of increases in healthcare costs. Grimmer et al. [6] found that costs of care were shown to decrease in 83% of the articles included. When specifically focusing on articles that examined CC in older adults, all of the articles reported a significant decrease in the cost of care. The benefits of the CC model may be limited to specific patient groups. Additionally, care plans that comply with CCS requirements in certain hospital settings have been perceived as challenging or unfeasible by some participants. Future research is needed to investigate both the impacts of CC in the acute care setting and the relationship between healthcare costs and the implementation of the CCS.

This study is the first survey of care professionals’ perceived knowledge, experiences, and perceptions of a national standard for CC. It contributes significantly to the understanding of the practical implementation and potential impacts of such a standard. The diverse composition of the sample, including care professionals from various disciplines and work settings across all Australian states and territories, ensures a comprehensive national perspective. The survey was developed through a robust process of preliminary pre-testing and pilot-testing, which effectively enhanced its face validity.

This study also has several limitations. The inclusion of only care professionals who had heard about the CCS may have also introduced a selection bias. Respondents may have self-selected, potentially leading to a bias where those with greater interest and understanding of the CCS were more inclined to participate. If so the knowledge and awareness of the CCS among care professionals would have been lower than indicated in this study. Results may be subject to recall bias regarding the implementation and impacts of the CCS as there is no baseline survey before the implementation of the CCS and our survey administration occurred five years after the release of the CCS. Further, the readiness and attitudes of care professionals may vary substantially owing to differences in baseline hospital procedures prior to the introduction of the CCS. Finally, an increased occurrence of missing data in the latter parts of the survey might suggest respondent fatigue or drop-out, possibly attributed to the survey’s length or limited understanding of CCS implementation, and therefore further exploration of care professionals’ perceptions about the CCS may be warranted to confirm these findings. However, despite these limitations, our study brings to light significant issues that should be taken into account to improve CCS implementation. It also serves as an important initial step in addressing knowledge gaps related to the implementation of a national standard for CC.

## Conclusion

Although Australian acute care hospitals have been mandated to implement the CCS, it is not easy to implement it successfully. Developing a CC plan is a key aspect of the CCS, yet developing these plans is challenging. Further education and training, resources, and collaboration may be required to increase care professionals’ capability and commitment to develop CC plans for patients. Nurses may benefit more from greater CCS education, as their knowledge of the CCS is lower than that of doctors, despite doing the bulk of CC delivery. More education and training as well as resources to support the development of MDT CC plans are needed to support the implementation of the CCS. Overall, more than half of care professionals felt that most care metrics had improved since the introduction of CCS but, almost half felt costs of care had also increased. Future research that involves investigating the implementation, costs and impacts of the CCS is warranted.

### Electronic supplementary material

Below is the link to the electronic supplementary material.

Supplementary Material 1

## Data Availability

The datasets generated and/or analysed during the current study are not publicly available due to privacy or ethical restrictions but are available from the corresponding author on reasonable request.

## Abbreviations

ACSQHC: Australian Commission on Safety and Quality in Health Care
CC: Comprehensive care
CCS: Comprehensive care standard
EMR: Electronic Medical Records
ieMR: Integrated Electronic Medical Records
MDT: Multidisciplinary team
NSQHS: National Safety and Quality Health Service
