# Supplementary material for: The implementation and impacts of the Comprehensive Care Standard in Australian acute care hospitals: a survey study
Source: BMC Health Serv Res. 2024 Jul 11;24:800. doi: 10.1186/s12913-024-11252-0 (PMC11241846; doi:10.1186/s12913-024-11252-0)

# Supplementary material

Table S1. Survey evaluation questions

| *Questions* | Responses | | |
| --- | --- | --- | --- |
| 1. Are the survey questions easy to read and understand? | Yes | No | *If No, why?* |
| 2. Are the response categories, layout and sequence adequate? | Yes | No | *If No, why?* |
| 3. Are the survey questions relevant to the Comprehensive Care Standard? | Yes | No | *If No, why?* |
| 4. Is the survey functional, easy to operate, practical to work through? | Yes | No | *If No, why?* |
| 5. Are any questions threatening or offensive? | Yes | No | *If Yes, why?* |

Table S2. Pilot test of the adequacy, relevance, practicability, simplicity, and ethicality of this survey

| Respondent No. | Simplicity | Relevance | Adequacy | Practicability | Ethicality |
| --- | --- | --- | --- | --- | --- |
| 1 | Yes | Yes | Yes | Yes | No |
| 2 | Yes | Yes | Yes | Yes | No |
| 3 | Yes | Yes | Yes | Yes | No |
| 4 | Yes | Yes | No | Yes | No |
| 5 | Yes | Yes | Yes | Yes | No |
| 6 | Yes | Yes | Yes | Yes | No |
| 7 | No | Yes | No | Yes | No |
| 8 | Yes | Yes | Yes | Yes | No |
| 9 | Yes | Yes | Yes | No | No |
| 10 | Yes | Yes | Yes | Yes | No |
| 11 | Yes | Yes | Yes | Yes | No |
| 12 | Yes | Yes | Yes | Yes | No |

Table S3. Main sections of the questionnaire on comprehensive care in acute care hospitals

| Knowledge | **How would you rate your knowledge of the Comprehensive Care Standard in terms of the** [**implementation guide**](https://www.safetyandquality.gov.au/our-work/comprehensive-care/comprehensive-care-tools-and-resources) **(the conceptual model and essential elements for delivering comprehensive care)?**  *(• none • very low • low •moderate • high • very high)* |
| --- | --- |
| Confidence | **How would you rate your confidence in performing the following essential elements associated with the Comprehensive Care Standard?**  *(•very low •low •moderate •high •very high)* |
|  | 1. ***Clinical assessment and diagnosis*** |
|  | 1. ***Identify goals of care*** |
|  | 1. ***Risk screening and assessment*** |
|  | 1. ***Develop a single comprehensive care plan*** |
|  | 1. ***Deliver comprehensive care*** |
|  | 1. ***Review and improve comprehensive care delivery*** |
| Support | **How would you rate the support available to you at your organisation in implementing the Comprehensive Care Standard in terms of the following areas?**  *(•very low •low •moderate •high •very high •not applicable)* |
|  | 1. ***Leadership across the organisation*** |
|  | 1. ***Education and training*** |
|  | 1. ***Equipment and tools*** |
|  | 1. ***System and process that support comprehensive care*** |
|  | 1. ***Standardisation of hospital practices and policy*** |
|  | 1. ***Ongoing quality improvement*** |
| Practice | **Did your organisation formally involve patients or care partners in the preparation, training, or implementation process of the Comprehensive Care Standard?**  *(• yes • no • don’t know)*  *If yes, how?* |
|  | **What is the proportion of patients in your area/unit that have a care plan that meets the CCS?**  *(• all • most • half • some • none • not applicable)*  *If not “all” or “not applicable”, why do you think this it is?* |
| Barriers | **Are there any challenges you are aware of at your organisation which interfere with implementing the Comprehensive Care Standard?**  *(• yes • no • don’t know)*  *If yes, what are the challenges?* |
| Facilitators | **Were there any things that were already in place that assisted in implementing the Comprehensive Care Standard at your organisation?**  *(• yes • no •don’t know)*  *If yes, what are the things?* |
| Perceived effects | ***How would you rate changes in patient outcomes following the introduction of the Comprehensive Care Standard at your organisation?***  *(•Decreased •No change •Increased •Not aware)* |
|  | 1. ***Interdisciplinary collaboration***   *Health care professionals from different disciplines working collaboratively and undertaking assessment, diagnosis, intervention, goal setting and care planning* |
|  | 1. ***Shared decision making***   *Patients, carers, and family members involved in discussions about their condition, prognosis, and care plan* |
|  | 1. ***Adverse events/clinical incidents***   *Event or circumstance that resulted, or could have resulted, in unintended and/or unnecessary harm to a person and/or a complaint, loss or damage* |
|  | 1. ***Psychological distress***   *Stress, anxiety, and depression relating to a hospital episode of care of both patient and family* |
|  | 1. ***Emotional/social/spiritual support***   *Emotional, Social and Spiritual Support patients and families received from health and social care professionals* |
|  | 1. ***Symptom control***   *Relief of distressing physical, emotional, social, and spiritual symptoms of both patient and family* |
|  | 1. ***Patient education***   *Specific and detailed educational activities and information provided to patients, carers, and family members so they can actively participate in decision making and care planning* |
|  | 1. ***Patient compliance***   *The extent to which the actual behaviour of the patient coincides with medical advice and instructions* |
|  | 1. ***Length of stay***   *The average duration of a single episode of hospitalisation* |
|  | 1. ***30-day readmissions***   *Patients who had been discharged from a hospital are admitted again within 30 days.* |
|  | 1. ***One-year survival***   *Patients alive 12 months after their hospital admission* |
|  | 1. ***Patient quality of life***   *Patients’ quality of life (ability to participate in or enjoy normal life events) from your perspective* |
|  | 1. ***Patient satisfaction***   *Patient satisfaction with the health care they received from your perspective* |
|  | 1. ***Health care costs***   *The costs for individuals, organisations, and a society directly or indirectly incurred by the provision of comprehensive care* |
|  | 1. ***Care continuity***   *Maintaining continuity of the medical care delivered to the patient when switching between caregivers or care institutions* |

Table S4. Perceived effects of the introduction of the Comprehensive Care Standard on patient care and health outcomes

| **Items** | ***n*** | **Unaware** | **Worsened** | **No change** | **Improved** |
| --- | --- | --- | --- | --- | --- |
| 1. *Interdisciplinary collaboration* | 397 | 21 (5.3) | 29 (7.3) | 114 (28.7) | 233 (58.7) |
| 1. *Shared decision making* | 398 | 22 (5.5) | 24 (6.0) | 122 (30.6) | 230 (57.8) |
| 1. *Adverse events/clinical incidents* | 401 | 42 (10.5) | 104 (25.9) | 129 (32.2) | 126 (31.4) |
| 1. *Psychological distress* | 398 | 41 (10.3) | 112 (28.1) | 129 (32.4) | 116 (29.1) |
| 1. *Emotional/social/spiritual support* | 394 | 34 (8.6) | 43 (10.9) | 129 (32.7) | 188 (47.7) |
| 1. *Symptom control* | 398 | 38 (9.5) | 48 (12.1) | 129 (32.4) | 183 (46.0) |
| 1. *Patient education* | 396 | 29 (7.3) | 35 (8.8) | 123 (31.1) | 209 (52.8) |
| 1. *Patient compliance* | 400 | 40 (10.0) | 37 (9.3) | 141 (35.3) | 182 (45.5) |
| 1. *Length of stay* | 393 | 51 (13.0) | 89 (22.6) | 134 (34.0) | 119 (30.3) |
| 1. *30-day readmissions* | 393 | 59 (15.0) | 94 (23.9) | 115 (29.3) | 125 (31.8) |
| 1. *One-year survival* | 399 | 78 (19.6) | 58 (14.5) | 113 (28.3) | 150 (37.6) |
| 1. *Patient quality of life* | 396 | 64 (16.2) | 32 (8.1) | 110 (27.8) | 190 (48.0) |
| 1. *Patient satisfaction* | 396 | 49 (12.4) | 36 (9.1) | 119 (30.0) | 192 (48.5) |
| 1. *Health care costs* | 400 | 68 (17.0) | 159 (39.7) | 113 (28.2) | 60 (15.0) |
| 1. *Care continuity* | 399 | 58 (14.5) | 39 (9.8) | 102 (25.6) | 200 (50.1) |

Table S5. Effects of the Introduction of the Comprehensive Care Standard on Patient Care and Health Outcomes by Demographics, Ranked in Descending Order of Improved Effects

|  | *gender* | *location* | *organisation* | *work unit* | *profession* | *leadership* | *experience* |
| --- | --- | --- | --- | --- | --- | --- | --- |
| 1. *Interdisciplinary collaboration* |  |  | *Y** |  |  |  |  |
| 1. *Shared decision making* | *Y** | *Y^*^#^ | *Y^* |  |  |  |  |
| 1. *Adverse events/ clinical incidents* |  |  |  |  |  |  | *Y* |
| 1. *Psychological distress* |  |  |  |  |  |  | *Y** |
| 1. *Emotional/social/ spiritual support* |  | *Y*^#^ |  |  | *Y** |  |  |
| 1. *Symptom control* |  | *Y*^* | *Y** |  |  |  |  |
| 1. *Patient education* | *Y** |  |  |  |  |  | *Y* |
| 1. *Patient compliance* | *Y** |  | *Y** | *Y^* | *Y** |  | *Y** |
| 1. *Length of stay* |  |  |  |  |  | *Y* | *Y^* |
| 1. *30-day readmissions* | *Y*^* |  | *Y** |  | *Y^* |  | *Y** |
| 1. *One-year survival* | *Y** |  |  |  | *Y** | *Y** | *Y** |
| 1. *Patient quality of life* | *Y** |  | *Y** | *Y^*^#^ |  |  | *Y** |
| 1. *Patient satisfaction* |  | *Y* |  |  |  |  | *Y** |
| 1. *Health care costs* |  | *Y^* |  |  |  | *Y* |  |
| 1. *Care continuity* |  |  |  |  |  |  | *Y* |

Y* significantly more "improved" outcome than “no change” outcome, Y^ significantly more “no change outcome” than “worsen” outcome, Y^#^ significantly more “improved” outcome than “worsen” outcome, Y an overall statistically significant difference exists; however, the pairwise comparisons of each category, using the Bonferroni correction, cannot pinpoint the specific categories that differ from each other.

Figure S1. Confidence in performing the six elements of the Comprehensive Care Standard

Figure S2. Support from healthcare organisations ranked in the descending order of very low to moderate support

**Questionnaire**

Note: Our questionnaire was conducted online, and the version presented below is the paper version that was used in the ethics application.


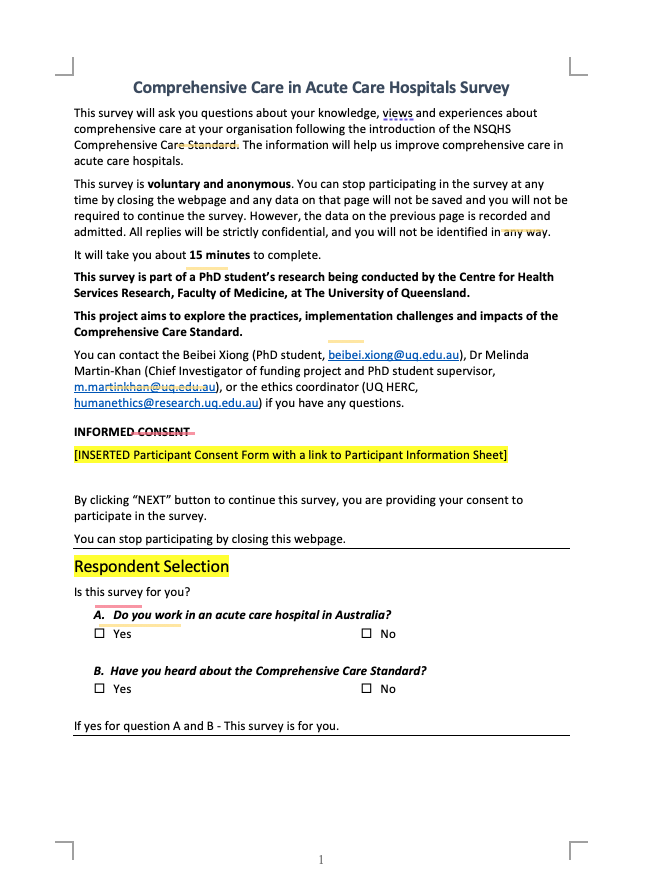


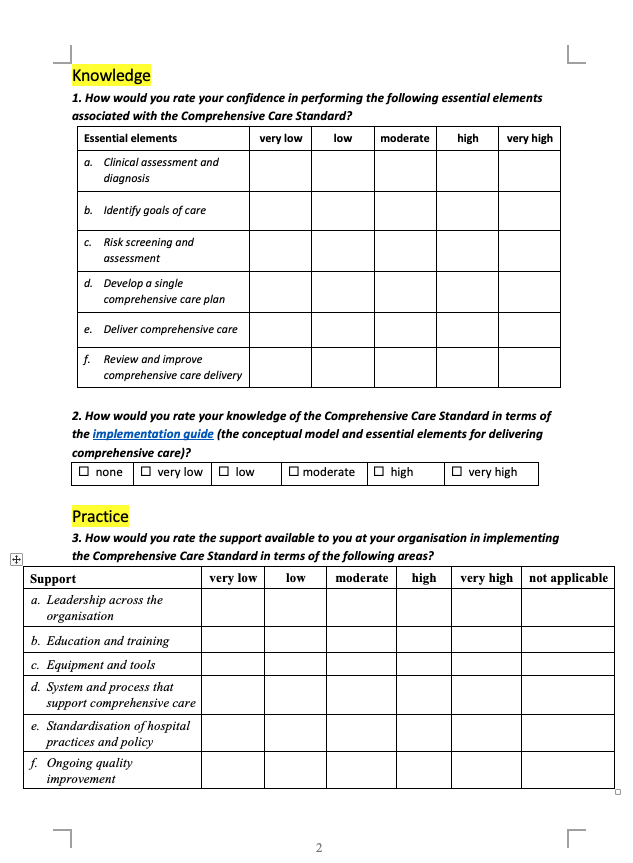


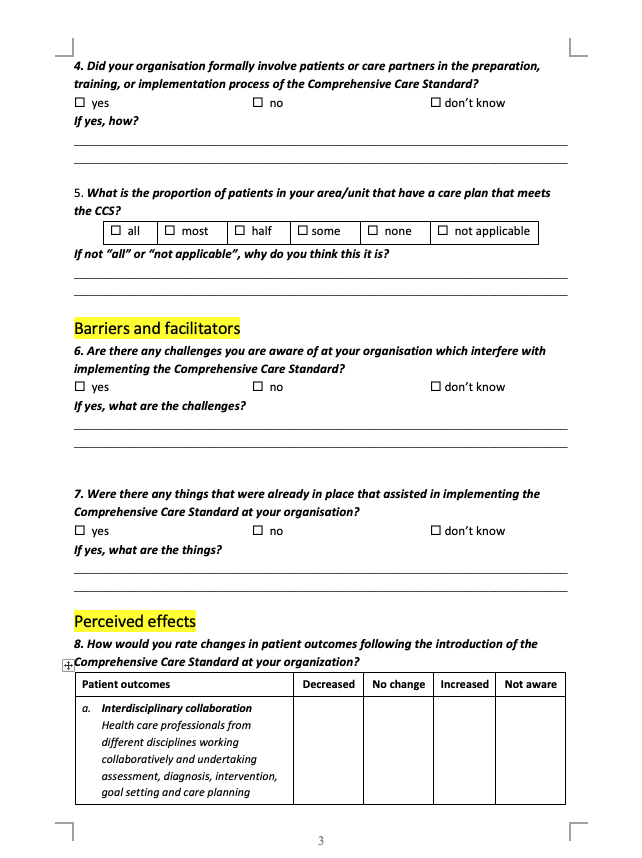


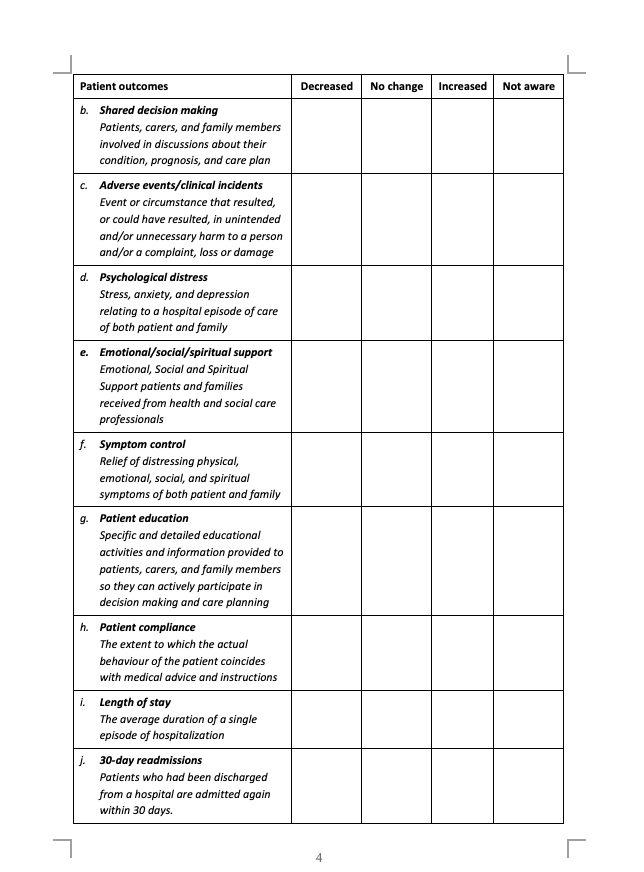


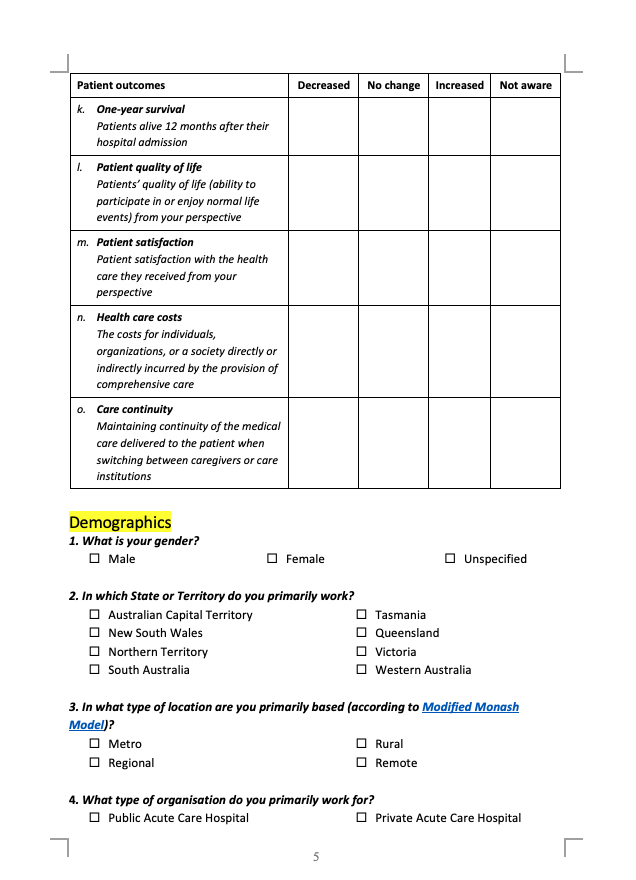


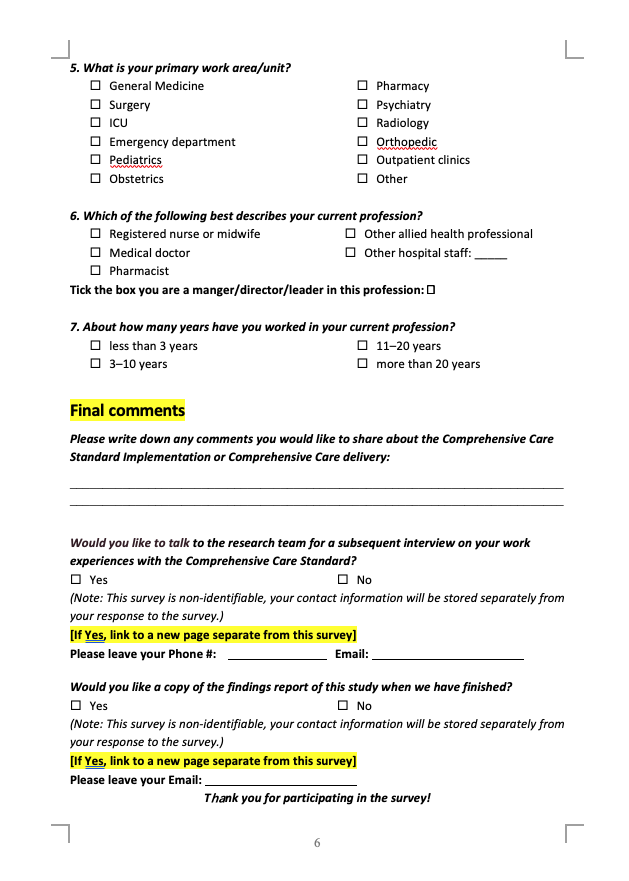

Supplement: Supplementary file 1 — Supplementary Material 1 [file 12913_2024_11252_MOESM1_ESM.docx]
